# Putting the brakes on centromere drive in *Mimulus*

**DOI:** 10.1371/journal.pgen.1009494

**Published:** 2021-04-22

**Authors:** Ching-Ho Chang, Harmit S. Malik

**Affiliations:** 1 Division of Basic Sciences, Fred Hutchinson Cancer Research Center, Seattle, Washington, United States of America; 2 Howard Hughes Medical Institute, Fred Hutchinson Cancer Research Center, Seattle, Washington, United States of America; University of Wisconsin–Madison, UNITED STATES

Asymmetric female meiosis in plants and animals is a battleground. Homologous chromosomes compete with each other for transmission through meiosis based on their centromeric DNA that recruits the chromosome segregation apparatus. Larger centromeres may recruit more centromeric proteins (centromere strength) and segregate into eggs more often than expected by mendelian inheritance (“centromere drive”) [1]. This subversion of female meiosis by “selfish centromeres” can be harmful and lower host fitness. Therefore, suppressors of centromere drive are predicted to arise, especially in centromeric proteins like CenH3/CENP-A. There is ample support for the first step of centromere drive [2–4], with one of the best demonstrations coming from studies in *Mimulus* monkeyflowers, in which a centromeric variant locus (***D***) undergoes centromere drive in interspecies and intraspecies crosses [2,5]. However, evidence of the second step of the model, i.e., suppression of centromere drive by centromeric proteins, remains elusive. A new study by Finseth and colleagues [6] dates the evolutionary origin of the ***D*** centromere to within the past 1,500 years of *Mimulus guttatus* evolution. It also reveals genetic variation in susceptibility to drive within *M*. *guttatus*, including a modifier of drive that maps to a CenH3 paralog, which adaptively evolved after the driving ***D*** centromere.

**Fig 1:**
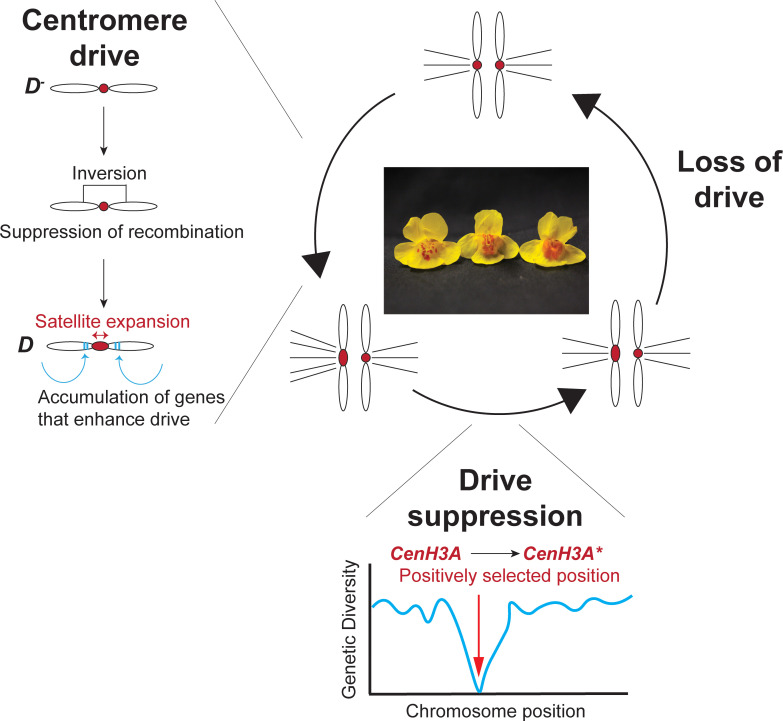
Three inferred stages of centromere drive and suppression in *Mimulus guttatus*. In the first stage (“Centromere drive”), a chromosome with non-driving centromeres (***D***^***−***^) acquires structural mutations, such as an inversion, and expansion of a centromeric satellite, which confers a transmission advantage in asymmetric female meiosis. Subsequently, it accumulates additional genes that enhance the drive, resulting in ***D***. Due to the ensuing costs to fertility, ***D*** chromosomes cannot fix in the population. In this scenario, suppressor alleles will arise in the second stage of this process (“Drive suppression”). One such suppressor allele may have arisen from a single amino acid variant of CenH3A. This allele underwent a selective sweep because it restored fitness in *M*. *guttatus* populations containing ***D*** chromosomes. Both driver and suppressor alleles are expected to coexist in populations due to frequency-dependent selection. Eventually, as suppressor alleles fix in populations, they negate the meiotic advantage of ***D*** chromosomes, which will then be lost and replaced by ***D***^***−***^ chromosomes in the third stage of the process (“Loss of drive”), resulting in fixed changes in CenH3A and potentially centromeric satellites.

A previous study had associated expanded centromere-specific repeats within ***D*** with its drive ability [2]. In the new study, Finseth and colleagues showed that half of Chromosome 11 (>12 Mb) is genetically linked to the selfish centromere on ***D*** (MDL11). Only 9 unique SNPs have accumulated in approximately 256,000 nucleotides of coding sequences across 13 distinct ***D*** lines. Based on this high similarity, the authors conclude that ***D*** arose within the last 1,000 to 1,500 years in *M*. *guttatus* after experiencing a recent selective sweep. In addition to an expanded array of repetitive satellite DNA, the MDL11 locus spans >387 protein-coding genes, including 45 genes unique to ***D*** chromosomes. It is likely that both centromeric repeats and linked genes contribute to drive (Fig 1).

*M*. *guttatus* ***D*** drives strongly against its counterpart centromere (***d***) from the self-fertilizing sister species *Mimulus nasutus* (***D*:*d*** = >98:2 [7]), and it drives weakly against intraspecies ***D***^***−***^ (***D*:*D***^***−***^ = 58:42 [2]). A trade-off between its drive ability and its cost to fertility maintains ***D*** at a frequency of 30% to 45% in *M*. *guttatus* populations [2,5]. Finseth and colleagues explored whether other genetic loci could influence the strength of ***D*** drive. Genetic loci linked to the drive locus should accumulate enhancers of drive, whereas unlinked genomic loci should accumulate suppressors of drive to restore genome fitness [8–10].

To identify modifiers of drive, Finseth and colleagues adopted an ingenious genetic mapping strategy. They tested drive of *M*. *guttatus* ***D*** against *M*. *nasutus* ***d*** or *M*. *guttatus* ***D***^***−***^ in distinct genetic backgrounds comprised of different genomic combinations of both species. Consistent with expectations, ***D*** drive against *M*. *nasutus* ***d*** was stronger than against *M*. *guttatus* ***D***^***−***^, with the strongest determinants of drive strength mapping to a closely linked region of MDL11. However, other genetic loci in the “mixed” genetic background also affected the strength of ***D*** drive. For example, in hybrid backgrounds, ***D/d*** drive was slightly weakened from 98:2 to 93:7, whereas ***D/D***^***−***^ drive was enhanced from 58:42 to 73:27. This meant that other genetic loci mapping outside of MDL11 in some *M*. *guttatus* genomes but absent in *M*. *nasutus* could suppress drive strength.

Further quantitative trait loci (QTL) mapping revealed 2 unlinked regions on Chromosome 9 and 14 associated with drive strength. The Chromosome 14 QTL locus center encodes one paralog of *CenH3* (named *CenH3A*), one of the leading candidates originally proposed as centromere drive suppressors [11]. Indeed, stratifying the data on *CenH3A* genotypes alone reveals a substantial contribution of this QTL on drive strength. Finseth and colleagues highlight a single segregating amino acid polymorphism in CenH3A that may contribute to its drive suppression properties. More precise genome editing will be required to directly test this prediction. Other analyses revealed that the *CenH3A* locus also experienced a recent selective sweep within the last 1,000 years after the selfish ***D*** centromere arose in *M*. *guttatus* populations [11]. For many drive systems, the ensuing cost is not necessarily caused by drive itself, but rather a result of linked deleterious mutations that accumulate due to suppression of recombination [10,12]. The finding that a *CenH3A* variant was positively selected in *Mimulus* populations exposed to centromere drive suggests that suppressing centromere drive is sufficient to restore fitness. These results provide strong support for a role of CenH3 proteins in affecting the strength of centromere drive.

The study also highlights the challenges in tracking drive amidst variable suppression in natural populations. Such suppressors could evolve contemporaneously with drive in situations like *Mimulus*, where the ***D*** centromere cannot become fixed in the population due to fitness costs. Alternatively, these suppressors could evolve subsequent to centromere drive going to fixation, as long as the costs of expanded centromeres remain manifest. In that respect, *Mimulus* is even more valuable as a model because it may have captured drive “in the act” where drive and suppressor alleles coexist and vie for supremacy in the same populations.

Cell biological studies of centromere drive in mice and *Drosophila* provide valuable clues to the molecular mechanism by which *CenH3A* alleles might suppress drive in the *Mimulus* system. For example, expansion of centromeric repeat satellites and increased recruitment of CenH3 proteins are directly associated with drive in mice [3]. By analogy, the driving ***D*** centromere might recruit more CenH3A relative to ***D***^***−***^ or ***d***. Conversely, CenH3A proteins from *M*. *guttatus* might have been selected to disfavor binding to ***D***, thereby suppressing centromere drive. Indeed, centromere-binding proteins with low centromeric affinity can weaken meiotic drive in mice [13]. The relative affinity of *Mimulus* CenH3A proteins for satellites between competing centromeres could explain why this new study found no straightforward hierarchy in *CenH3A* allele combinations and drive suppression in the new study. This difference could be the result of relative affinity differences between CenH3A variants for satellite DNA. Alternatively, this could be a result of protein–protein interactions. Indeed, Finseth and colleagues found that the CenH3 chaperone NASP^Sim3^ [14] is closely linked to the driving ***D*** centromere. Allelic differences in NASP^Sim3^ could directly affect chaperone:CenH3A binding affinity and ultimately CenH3A deposition, like they do in *Drosophila* [15]. Some of these predictions will be testable in future experiments via cytological localization and biochemical affinity tests of the various CenH3A variants. Such experiments will establish the *Mimulus* system both as a premier ecological as well as cell biological model of centromere drive.
